# Foxp3 is correlated with VEGF-C expression and lymphangiogenesis in cervical cancer

**DOI:** 10.1186/s12957-017-1221-5

**Published:** 2017-09-18

**Authors:** Jiabu Tang, Zheng Yang, Zhuo Wang, Zhen Li, Hongmei Li, Jinbao Yin, Min Deng, Wei Zhu, Chao Zeng

**Affiliations:** 1 Department of Pathology, Guangdong Medical University, 1 Xincheng Road, Dongguan, 523808 China; 2 Department of Pathology, The First Affiliated Hospital of Sun Yat-sen University, Zhongshan 2nd Road 74, Guangzhou, 510080 China; 3 Cancer Hospital and Cancer Research Institute, Guangzhou Medical University, Guangzhou, 510095 China

**Keywords:** Foxp3, Cervical cancer, Lymphangiogenesis

## Abstract

**Background:**

Recent observations revealed Foxp3 participated in the development of cervical cancer. Furthermore, Foxp3 has a vital function in the lymphatic metastasis of cervical cancer. However, it is unclear whether Foxp3 is correlated with lymphangiogenesis of cervical cancer.

**Methods:**

In this experiment, expression of Foxp3 and VEGF-C was detected in 50 cervical cancer samples by immunohistochemistry. In addition, we evaluated the association between Foxp3 and VEGF-C expression and lymphangiogenesis of cervical cancer evaluated by lymphatic vessel density.

**Results:**

These data demonstrate Foxp3 is positively correlated with VEGF-C expression. Furthermore, Foxp3 is associated with lymphangiogenesis of cervical cancer.

**Conclusions:**

These results revealed Foxp3 play an important role in lymphangiogenesis of cervical cancer.

**Trial registration:**

Gunagdong Medical University, PJ2013049

## Background

Cervical cancer is the second common malignant tumor in female [1]. As cervical cancer is characterized by few clinical manifestations, it is hard to detect their existence in early stage. Hence, the prognosis of cervical cancer in advanced stage is unsatisfactory. A well-defined etiology of cervical cancer is infected by high-risk human papillomavirus (HPV). However, the underlying pathogenesis of the disease requires further research.

FOXP3, a member of a forkhead box proteins family, contained a winged helix DNA-binding domain. Human Foxp3 gene is located at Xp1 1.23, and it plays as a controller of the function of regulatory T-cells, which have a vital role in the process of forming microenvironment with immunosuppressive tumor [2–7]. Recent studies report Foxp3 is not only presented in Treg cells but also expressed in a variety of cancer cells [8–16]. For example, Foxp3 is over-expressed in breast [12], gastric [14], and thyroid cancer [13], and it also closely correlated with progression and prognosis of these cancers. Luo et al. revealed Foxp3 participated in advancement of normal cervical tissues to cervical cancer [17]. Furthermore, Foxp3 had a significant role in facilitating lymphatic metastasis of cervical cancer [17, 18], and high Foxp3 expression in the cervical cancer predicted a poor prognosis [17].

Recently, Sasahira et al. revealed Foxc2 regulated angiogenesis and lymphangiogenesis in oral squamous cell carcinoma [19]. Foxc1 and Foxc2 are required for lymphatic sprouting during vascular development [20]. Nevertheless, until now, no research has reported the association of Foxp3 expression with lymphangiogenesis of cervical cancer. In this experiment, we investigated the association of Foxp3 with VEGF-C expression and the role of Foxp3 in lymphangiogenesis of cervical cancer.

## Methods

### Patients and tissue samples

In the assay, 50 cervical cancer samples were obtained from un-selected patients at Department of Pathology, the First Affiliated hospital of Sun Yat-sen University. More details of clinical and pathological information about these patients are listed in Table 1. In these samples, 42 cases were squamous cancer and 8 cases were adenocancer. Thirty-four cases had no lymph node metastasis, and 16 cases present lymph node metastasis. The study was approved by Institutional Research Ethics Board of Guangdong Medical University.

**Table 1:** Correlation of clinicopathological parameters with Foxp3 and VEGF-C in cervical cancer patients

Clinical parameter	Foxp3 expression		*P* value	VEGF-C expression		*P* value
	Positive (%)	Negative (%)		Positive (%)	Negative(%)	
Age (year)						
< 50	14 (28)	10 (20)		20 (40)	4 (8)	
≥ 50	19 (38)	7 (14)	0.373	18 (36)	8(16)	0.327
Differentiation						
High + moderate	17(34)	8 (16)		23 (46)	4 (8)	
Low	16(32)	9 (18)	1.000	15 (30)	8(16)	0.183
Tumor size						
< 4 cm	12 (24)	9 (18)		16 (32)	5 (10)	
≥ 4 cm	21 (42)	8 (16)	0.366	22 (44)	7 (14)	0.624
Histological type						
Squamous carcinoma	30 (60)	12 (24)		34 (68)	8(16)	
Adenocarcinoma	3 (6)	5 (10)	0.102	4 (8)	4 (8)	0.082
Clinical stage						
I + II	19(38)	16 (32)		30(60)	5(10)	
III + IV	14(28)	1(2)	0.009	8 (16)	7 (14)	0.027
Lymph node metastasis						
Absent	19(38)	15(30)		23 (46)	11(22)	
Present	14(28)	2(4)	0.026	15 (30)	1 (2)	0.042

### Immunohistochemistry

Paraffin block was cut into 4-μm sections and treated by routine skill. After microwaved in citrate buffer for 5 min, the slides were incubated with Foxp3 (ab10563, Abcam, USA), VEGF-C (sc-374,628, Santa Cruz, CA), and D2-40 (MAB-0567, Maxim-Bio, Fuzhou, China) at room temperature respectively. Then, the sections were incubated with a secondary antibody (MaximBio Company, Fuzhou, China). Labeling was monitored by diaminobenzidine (Maxim-Bio Company). At last, hematoxylin was used to stain the sections.

### IHC evaluation

Foxp3 and VEGF-C expression was scored in accordance with the intensity (0, no staining; 1, weak staining; 2, moderate staining; 3, strong staining) and extent staining of cervical cancer cells that were stained (0, no stained; 1, < 10% cervical cancer cells stained; 2, 10–50% cervical cancer cells stained; 3, > 50% cervical cancer cells stained; 4, > 75% cervical cancer cells stained). If the data of multiplication between staining intensity and the extent of positive cervical cancer cells is ≥ 2, it is regarded as positive (+). Finally, in five unselected areas of a 1-mm^2^ field, the number of lymphatic vessels was accumulated, then calculated the average.

### Statistical analysis

All statistical analyses were done by SPSS 13.0 (SPSS, Inc., Chicago, IL). *χ* ^2^ test was used to evaluate the association of Foxp3 and VEGF-C with clinicopathological parameters. The relation of Foxp3 with VEGF-C was evaluated by Fisher’s exact. The difference was statistical when the *P* value is < 0.05.

## Results

### Expression of Foxp3 and VEGF-C in cervical cancer

**Fig. 1:**
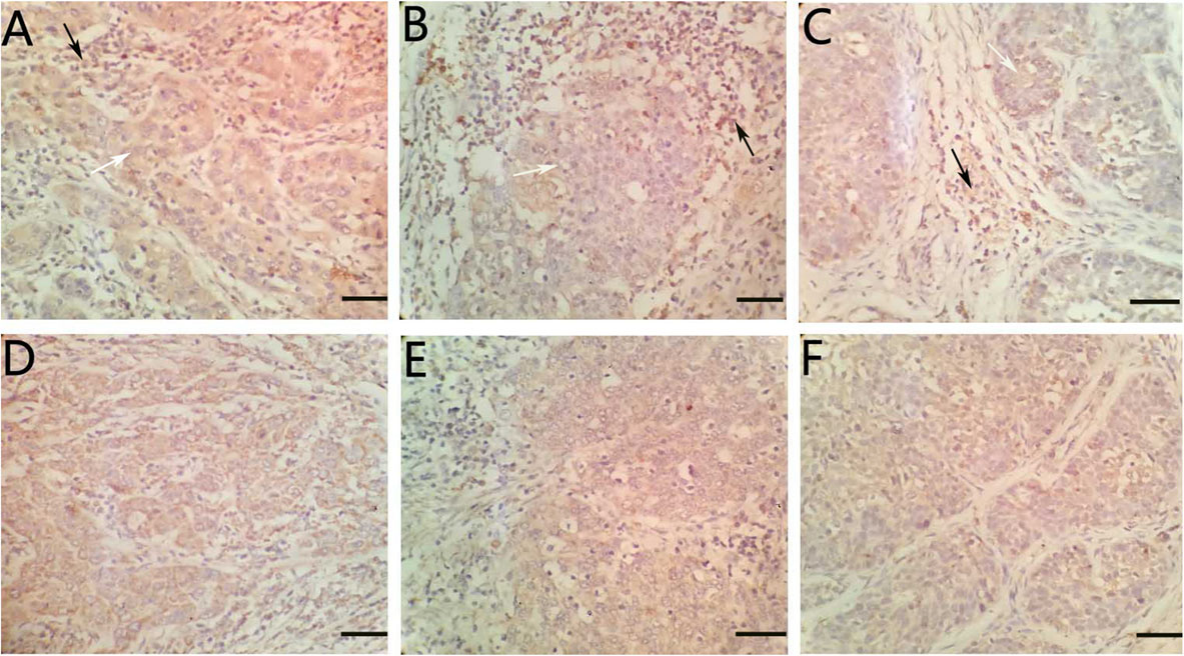
Immunohistochemical staining of Foxp3 and VEGF-C in cervical cancer tissues. **a**–**c** Positive Foxp3 expression in cervical cancer cells (*white arrowheads*) and positive Foxp3 expression in lymphocytes (*black arrowheads*). **d**–**f** Positive VEGF-C expression in cervical cancer (×100, scale bar 50 μm)

**Fig. 2:**
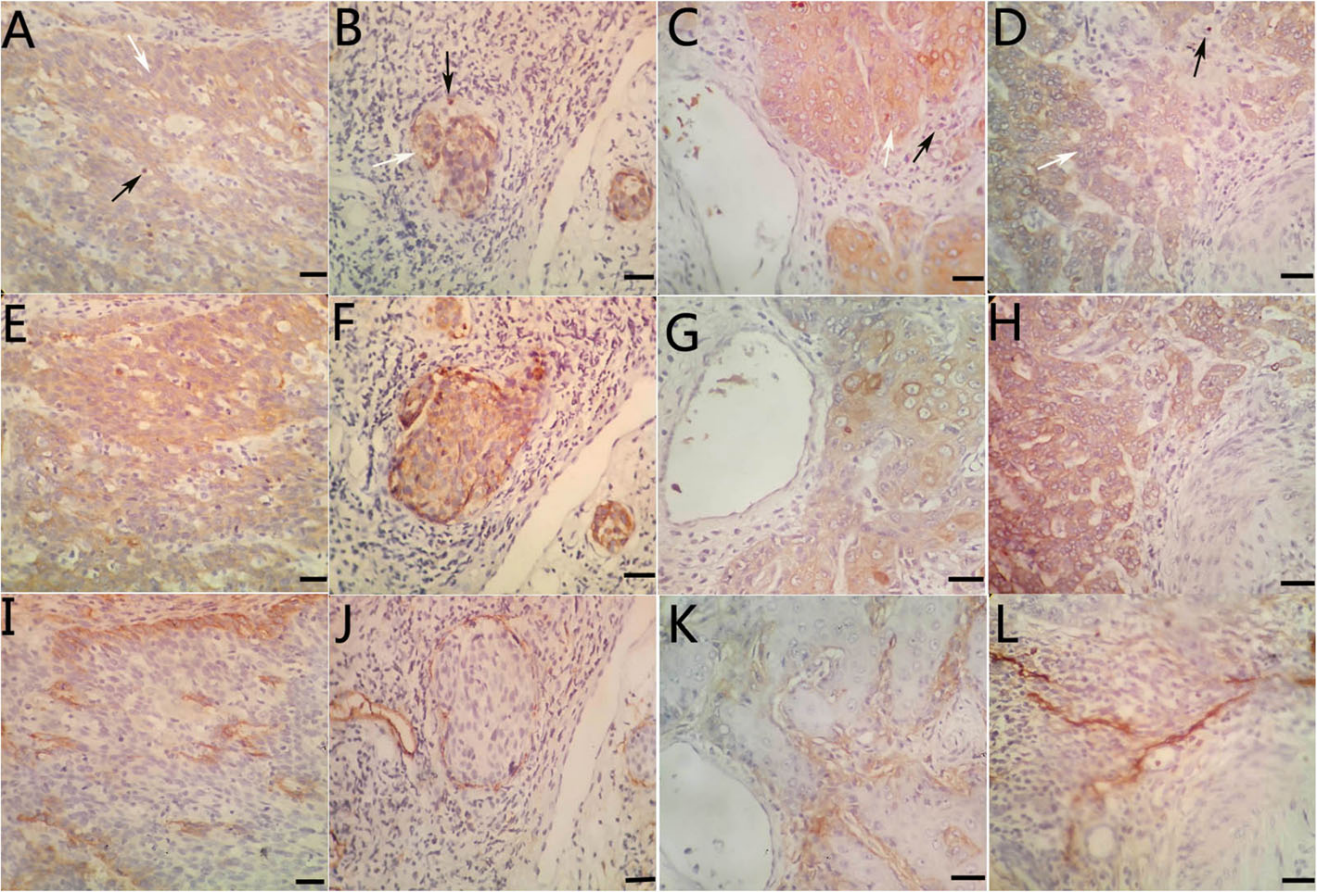
Representative photomicrographs of immunohistochemical staining of Foxp3 (**a**–**d**; *white arrowheads*: positive Foxp3 expression in cervical cancer cells; *black arrowheads*: positive Foxp3 expression in lymphocytes), VEGF-C (**e**–**h**) and lymphatic vessels (**i**–**l**) (×200, scale bar 50 μm)

Immunohistochemical staining of Foxp3 was performed in 50 cervical cancer cases. After evaluating by two pathologists, Foxp3 expression was observed in 66% (33/50) cervical cancer tissues. As shown in Fig. 1a–c and Fig. 2a–d, Foxp3 was found not only in nuclear of lymphocytes but also in cytoplasm of cervical cancer cells. Similarly, VEGF-C was also immunostained in the cytoplasm of the cervical cancer cells (Fig. 1d–f; Fig. 2e–h).

Table 1 demonstrates the association between Foxp3 expression and clinicopathological factors. Expression of Foxp3 was positive in 87.5% (14/16) cases that had lymph node metastasis, and the positive rate was significantly higher than the samples with the absence of lymph node metastasis (55.9%, 19/34). Furthermore, statistical analysis demonstrates that Foxp3 immunoreactivity is associated with some clinicopathological factors, such as clinical stage and lymph node metastasis (all *P* < 0.05). However, there was no significant relation between Foxp3 expression and the other clinical parameters, including age, tumor size, and histological type (all *P* > 0.05). On the other hand, VEGF-C immunoreactivity was observed in 76.0% samples (38/50). As suggested in Table 1, VEGF-C expression had a significant association with cases that presented lymph node metastasis (*P* < 0.05).

### Comparison of Foxp3 and VEGF-C expression

Both Foxp3 and VEGF-C had immunoreactivity in 31 samples. On the other hand, neither Foxp3 nor VEGF-C was expressed in 10 samples. Only Foxp3 was expressed in 2 samples, whereas VEGF-C alone was expressed in 7 samples (Table 2). These results indicated that Foxp3 expression was significantly associated with VEGF-C expression (*P* < 0.05)**.**

**Table 2:** Association of Foxp3 with VEGF-C expression

	Foxp3 (−)	Foxp3 (+)	*P*
VEGF-C (−)	10	2	
VEGF-C (+)	7	31	< 0.0001

### Expression of Foxp3 and VEGF-C predicts lymphangiogenesis of cervical cancer

In this experiment, D2-40 was utilized to assess lymphatic vessel density (LVD) in cervical cancer tissues. As shown in Table 3, Foxp3-positive cases had more lymphatic vessel than Foxp3-negative cases (11.25 ± 3.16 VS 4.14 ± 2.76). Similarly, cases with VEGF-C staining had more lymphatic vessel compared with cases that had no VEGF-C expression (14.67 ± 4.52 vs 5.32 ± 2.27). Notably, samples that combined with Foxp3 expression and VEGF-C expression had the highest lymph vessel (16.83 ± 5.29; Fig. 2).

**Table 3:** Mean value of LVD according to expression patterns of Foxp3 and VEGF-C

	LVD	*P*
Foxp3 (+)	11.25 ± 3.16	
Foxp3 (−)	4.14 ± 2.76	< 0.05
VEGF-C (+)	14.67 ± 4.52	
VEGF-C (−)	5.32 ± 2.27	< 0.001

## Discussion

Foxp3, a member of the FOX protein family, is a forkhead (FKH) box transcription factor. It contains a DNA-binding FKH box domain which plays as a transcriptional activator and repressor of specific genes. As known to all, Foxp3 is widely known for its function in the development of immunoregulatory T cells [21]. Recently, it has been discovered that abnormal Foxp3 expression is associated with a series of cancers, such as prostate [22], ovary [16], and breast cancer [12].

Extrinsic expression of Foxp3 inhibits proliferation and induces apoptosis of gastric cancer cells by activating ADP-ribose polymerase1 (PARP), caspase-3 and caspase-9 [23]. Moreover, Tan et al. reported Foxp3 over-expression significantly reduced the proliferation of melanoma cells in vitro and in vivo [8]. These findings indicated that Foxp3 might act as a tumor suppressor gene. Conversely, high Foxp3 expression of the colorectal cancer cells was correlated with unfavorable prognosis compared with cases that had low Foxp3 expression [24]. Similarly, our data suggested Foxp3 expression is correlated with higher clinical stage and lymph node metastasis. This result demonstrated Foxp3 might be involved in lymph node metastasis of cervical cancer.

Interestingly, FOXP3 expression in breast cancer cells was correlated with high Ki-67 index, indicating high proliferative activity of FOXP3-positive tumors. Merlo et al. also demonstrated increased Ki-67 staining in FOXP3-mutated mammary cells. These results indicated that FOXP3 expression could promote proliferation of cancer cells. However, in our study, there was no relation between Foxp3 expression and tumor size. This discrepancy is attributed to different tumor types or absence of cell experiment.

The current study, for the first time, revealed the role of Foxp3 in lymphangiogenesis of cervical cancer. Firstly, we found Foxp3-positive cases had more lymphatic vessel than Foxp3-negative cases. VEGF-C is essential for most lymphangiogenic processes by activating the vascular endothelial growth factor receptors VEGFR-3 and VEGFR-2 [25]. Then, taking into account the critical role of VEGF-C in lymphangiogenesis, we evaluated the correlation between Foxp3 and VEGF-C expression. In this study, our findings suggest Foxp3 had a significant positive correlation with VEGF-C in cervical cancer.

## Conclusions

The present assay shows that Foxp3 expression is associated with advancement of cervical cancer and lymph node metastasis. More importantly, Foxp3 might promote lymphatic vessel formation in cervical cancer correlated with VEGF-C expression. Although further studies are needed to elucidate the molecular mechanism of Foxp3 in lymphangiogenesis of cervical cancer, the present assay will provide new insights into the lymph node metastasis of cervical cancer.

## Abbreviations

HPV: High-risk human papillomavirus
LVD: Lymphatic vessel density
